# Supplementary material for: CD4+ and CD8+ regulatory T cell characterization in the rat using a unique transgenic Foxp3-EGFP model
Source: BMC Biol. 2023 Jan 12;21:8. doi: 10.1186/s12915-022-01502-0 (PMC9837914; doi:10.1186/s12915-022-01502-0)
Supplement: Supplementary file 1 — Additional file 1: Supplementary figure 1. Flow cytometry analyses of spleen and thymus cells from Foxp3-EGFP rats. A) Spleen, thymus and bone marrow were harvested from 12 weeks-old Foxp3-EGFP or wild-type (WT) rats and single cell suspensions were gated by SSC and FSC on lymphocytes followed by the identification with mAbs of major cell populations such as TCR+ cells (TCR+CD4+, TCR+CD8+), TCR− cells (TCR-CD4+ and TCR-CD8+) and CD161+ for NK cells. These populations were then analyzed for FOXP3 expression by EGFP expression and by using an anti-FOXP3 mAb. Contour plots from one animal representative of 6 analyzed in the same conditions. Right hand graphs are the mean and SEM of all animals analyzed. Student’s t test *P < 0.05, **P < 0.01, ***P < 0.001, and ****P < 0.0001. B) PBMCs from WT and Foxp3−EGFP+ rats were analyzed using untreated (left panels) or permeabilized and fixed (2 middle panels) cells with the conditions used for the analysis using anti-FOXP3 antibodies. Compared to untreated cells, EGFP signals were reduced in permeabilized cells, as previously described [59]. Co-labeling using anti-FOXP3 antibodies and EGFP in permeabilized cells (2 right panels) showed co-staining in CD4+ and CD8+ T cells from Foxp3-EGFP animals. C. EGFP+ cells were sorted from spleen, permeabilized and fixed followed by analysis using anti-FOXP3 antibodies. All sorted EGFP+ cells expressed FOXP3. Supplementary figure 2. Suppression assay using T CD4+EGFP+ and CD8+EGFP+ cells. Representative histograms of a suppressive assay using spleen cells from 12 weeks-old Foxp3-EGFP rats. TCR+CD4+CD25+CD127lowEGFP+ (labeled with CPD-450) responder cells and TCR+CD8+CD45RClow/−EGFP+ cells were sorted and added to an MLR in a 1:1 or 1:0.5 Tresponder to Tregs cells. The MLR was performed by co-culturing in a 1:1 ratio spleen CD4+CD25− Tconv cells from SPD rats (MHC haplotype u) labeled with CPD-670 along with enriched spleen APCs from Lewis 1A (MHC haplotype a) rats. Decrease in the percenta [file 12915_2022_1502_MOESM1_ESM.docx]

**Additional File1: Table S1. Frequency of CD4^+^EGFP^+^ and CD8^+^EGFP^+^ in *Foxp3-EGFP* rats and *Foxp3-EGFP* mice.**

**A**

|  |  | **LN** | **spleen** | **BM** | **blood/ml** |
| --- | --- | --- | --- | --- | --- |
| **TCR+CD4+EGFP+** | **Rat** | **7.8 ± 0.8** | **10.4 ± 1.2** | **8.9 ± 1.2** | **8.2 ± 1.0***** |
|  | **Mouse** | **3.9 ± 1.5** | **9.7 ± 1.1** | **7.9 ± 1.8** | **3.7 ± 0.5***** |
| **TCR+CD8+EGFP+** | **Rat** | **1.2 ± 0.19** | **1.1 ± 0.28*** | **1.24 ± 0.41** | **0.69 ± 0.07** |
|  | **Mouse** | **0.3 ± 0.3** | **0.3 ± 0.09*** | **0.06± 0.04** | **0.4 ± 0.14** |

**n=6 for rats and n=5 for mice**

**mean ± SEM, *p-value < 0.05 and ***p-value < 0.001**


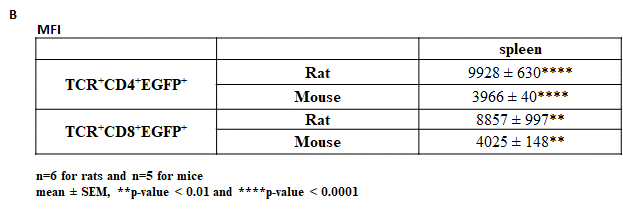


**Additional File1: Table S2. Immune genes upregulated in TCD4+EGFP+ vs. TCD4+EGFP- cells.**

**TCD4+EGFP+ TCD4+EGFP-**

| **Name of gene** | **Log 2 fold change** | **Name of gene** | **Log 2 fold change** |
| --- | --- | --- | --- |
| ***Itgb8*** | **4.7371** | *Il17rc* | -4.8380 |
| ***Tnfrsf13b*** | **4.2417** | *Il17re* | -4.1888 |
| ***Foxp3*** | **3.5868** | *Sphk1* | -3.6848 |
| ***Lrrc32*** | **3.2384** | *Ccr6* | -2.3299 |
| ***Cd80*** | **2.8669** | *Klrc1* | -2.0664 |
| ***Ccn3*** | **2.8574** | *Cd40lg* | -1.8672 |
| ***Ikzf2*** | **2.8187** | *Map3k6* | -1.7279 |
| ***Stap2*** | **2.5447** | *Lgals3* | -1.6431 |
| ***Il1rl1*** | **2.4686** | *Tnf* | -1.4869 |
| ***Ptafr*** | **2.3347** | *Gzmc* | -1.2828 |
| ***C1qtnf6*** | **2.2820** | *Tnfrsf21* | -1.2513 |
| ***Cbx6*** | **2.1543** | *Il17ra* | -1.2335 |
| ***Tox*** | **1.9271** | *Foxm1* | -1.2177 |
| ***Prf1*** | **1.9247** | *Cd226* | -1.2115 |
| ***Fcmr*** | **1.9183** | *Cd7* | -1.1829 |
| ***Lbh*** | **1.8573** | *Il3ra* | -1.1256 |
| ***Gpr15*** | **1.7579** | *Klrd1* | -1.0954 |
| ***Slamf7*** | **1.7443** | *Il17f* | -1.0861 |
| ***Il2rb*** | **1.7114** | *Mcoln2* | -1.0473 |
| ***Vipr1*** | **1.6450** | *Prr5l* | -1.0335 |
| ***Nrp1*** | **1.5586** |  |  |
| ***Art2b*** | **1.5327** |  |  |
| ***Peli1*** | **1.5056** |  |  |
| ***Aff3*** | **1.4599** |  |  |
| ***Tnfrsf9*** | **1.3500** |  |  |
| ***Igf1r*** | **1.3340** |  |  |
| ***Cradd*** | **1.3260** |  |  |
| ***Map3k1*** | **1.2793** |  |  |
| ***Cd9*** | **1.2674** |  |  |
| ***Prkcz*** | **1.2653** |  |  |
| ***Gata3*** | **1.2224** |  |  |
| ***Rab20*** | **1.2077** |  |  |
| ***Irak3*** | **1.1809** |  |  |
| ***Cd38*** | **1.1486** |  |  |
| ***Sell*** | **1.1299** |  |  |
| ***Adora2a*** | **1.1244** |  |  |
| ***Pvr*** | **1.0878** |  |  |
| ***Fas*** | **1.0522** |  |  |
| ***Il4r*** | **1.0304** |  |  |
| ***Il18*** | **1.0003** |  |  |

adjusted p-value<0.05

**Additional File1: Table S3. Immune genes upregulated in TCD8+EGFP+ vs. TCD8+EGFP- cells.**

**TCD8+EGFP+ TCD8+EGFP-**

| **Name of gene** | **Log 2 fold change** | **Name of gene** | **Log2 fold change** |
| --- | --- | --- | --- |
| *C1qc* | 4.4393 | *Gzmk* | -4.7022 |
| *Foxp3* | 4.1674 | *Ly49s3* | -4.6432 |
| *C1qa* | 3.7324 | *Ccl5* | -3.3444 |
| *Fcna* | 3.7230 | *Cx3cr1* | -3.1771 |
| *Spic* | 3.3882 | *LOC688090* | -2.9252 |
| *Pla2g2d* | 3.2813 | *Nkg7* | -2.6903 |
| *Hmox1* | 3.2730 | *Ccl6* | -2.5610 |
| *C1qb* | 3.2507 | *Gzma* | -2.5162 |
| *Lrrc32* | 3.2369 | *Ly49s6* | -2.3763 |
| *Vcam1* | 3.1587 | *Gzmm* | -2.2315 |
| *Cdh1* | 3.0837 | *Gzmc* | -2.2299 |
| *Laptm4b* | 2.9295 | *S1pr5* | -2.2168 |
| *Cd163* | 2.8150 | *Slamf7* | -2.0638 |
| *Erc1* | 2.8017 | *Def6* | -2.0232 |
| *Mrc1* | 2.8002 | *Susd2* | -1.9913 |
| *Dnase1l3* | 2.7207 | *Cst7* | -1.9721 |
| *Kdr* | 2.7139 | *Dapk2* | -1.9215 |
| *Maf* | 2.6682 | *Klre1* | -1.9205 |
| *Trpm2* | 2.3964 | *Pycard* | -1.7903 |
| *Fcmr* | 2.3651 | *Ctsw* | -1.7737 |
| *Lgmn* | 2.3513 | *Serpinb9* | -1.7531 |
| *Itgb5* | 2.3448 | *Fcnb* | -1.7273 |
| *Blnk* | 2.2912 | *Cxcr6* | -1.6758 |
| *Cadm4* | 2.2339 | *Myadm* | -1.6118 |
| *Cd5l* | 2.1863 | *RT1-DMa* | -1.5824 |
| *Mzb1* | 2.1551 | *Faslg* | -1.5775 |
| *Cfd* | 2.1034 | *Plek* | -1.5573 |
| *Rarg* | 2.0133 | *Klrb1a* | -1.5202 |
| *Anxa3* | 1.9852 | *S100a9* | -1.4528 |
| *Il9r* | 1.9399 | *Lgals3bp* | -1.4337 |
| *Aff3* | 1.8397 | *Ncr1 (CD335)* | -1.4086 |
| *Il2ra* | 1.8367 | *Klra22* | -1.3510 |
| *Tnfrsf11b* | 1.7848 | *RT1-Da* | -1.3030 |
| *Phlda1* | 1.7846 | *Klrk1 (KKG2-D)* | -1.2803 |
| *Cd79b* | 1.7722 | *Cd38* | -1.2502 |
| *Ly49s7* | 1.7662 | *Klrd1* | -1.2018 |
| *Jag1* | 1.7608 | *RT1-Db1* | -1.1809 |
| *Pou2f2* | 1.7478 | *Cd59* | -1.1539 |
| *Dclre1c* | 1.7469 | *Cxcr3* | -1.1520 |
| *C1qtnf6* | 1.7104 | *RT1-DMb* | -1.1539 |
| *Tmem176b* | 1.6492 | *Fyn* | -1.1043 |
| *Timp1* | 1.6283 | *Nmi* | -1.0948 |
| *Tmem176a* | 1.6218 | *Cd3g* | -1.0827 |
| *Irak3* | 1.6048 | *Cd8b* | -1.0764 |
| *Ctsl* | 1.5348 | *Tnfsf9* | -1.0733 |
| *Cfp* | 1.5120 | *Sh2d1a* | -1.0703 |
| *Il6st* | 1.4227 | *Cd2* | -1.0583 |
| *Tnfrsf9* | 1.3808 | *Casp3* | -1.0473 |
| *Art2b* | 1.3502 | *Cd74* | -1.0352 |
| *Lta* | 1.3047 |  |  |
| *Il4r* | 1.2441 |  |  |
| *Bcl3* | 1.2323 |  |  |
| *Socs1* | 1.1844 |  |  |
| *Vipr2* | 1.1346 |  |  |
| *Tfrc (CD71)* | 1.1160 |  |  |
| *Il6r* | 1.0884 |  |  |
| *Bcl2* | 1.0220 |  |  |
| *Ikbke* | 1.0057 |  |  |

adjusted p-value <0.05

**Additional File1: Table S4. Immune genes upregulated in CD4+EGFP+ vs. CD8+EGFP+ cells.**

**TCD4+eGFP+ TCD8+eGFP+**

| **Name of gene** | **Log2 fold change** | **Name of gene** | **Log2 fold change** |
| --- | --- | --- | --- |
| ***LOC688090*** | **3.4725** | *Fcna* | -2.5401 |
| ***Cd4*** | **3.2752** | *Klri1* | -2.4753 |
| ***Ms4a1*** | **3.0906** | *Cd7* | -2.2708 |
| ***Cd300c2*** | **2.7454** | *Cd8b* | -2.0966 |
| ***Ccl6*** | **2.5616** | *Hmox1* | -2.0702 |
| ***Selplg*** | **2.4561** | *C1qc* | -2.0702 |
| ***Nrp1*** | **2.2765** | *C1qa* | -2.0194 |
| ***Gata3*** | **2.1798** | *Cd8a* | -1.9326 |
| ***Ptafr*** | **2.1467** | *Spic* | -1.9273 |
| ***Fcmr*** | **2.0031** | *Cdh1* | -1.8467 |
| ***Lbh*** | **1.9287** | *C1qb* | -1.8234 |
| ***Fcnb*** | **1.8856** | *Dnase1l3* | -1.7839 |
| ***Cd79b*** | **1.8688** | *Lag3* | -1.4806 |
| ***Spib*** | **1.8052** | *Cxcl17* | -1.4593 |
| ***Ly86*** | **1.7394** | *Cfd* | -1.4120 |
| ***Ccr6*** | **1.6931** | *Tbx21* | -1.3325 |
| ***RGD1559482*** | **1.6797** | *Mcoln2* | -1.2776 |
| ***Cd19*** | **1.5346** | *Il17ra* | -1.2213 |
| ***Gpx1*** | **1.3639** | *Cxxc5* | -1.2136 |
| ***Rab25*** | **1.3533** | *Gzmm* | -1.1978 |
| ***Ctsc*** | **1.2477** | *Tnk2* | -1.1217 |
| ***Cd74*** | **1.2387** | *Ptms* | -1.0519 |
| ***Tnfrsf4*** | **1.1885** |  |  |
| ***Isg15*** | **1.1781** |  |  |
| ***Pvr*** | **1.1735** |  |  |
| ***Anxa1*** | **1.1715** |  |  |
| ***Lrrc32*** | **1.1595** |  |  |
| ***Tapbp*** | **1.1323** |  |  |
| ***Skap2*** | **1.1203** |  |  |
| ***Icos*** | **1.0982** |  |  |
| ***Nmi*** | **1.0799** |  |  |
| ***RT1-Da*** | **1.0756** |  |  |
| ***Ctsl*** | **1.0504** |  |  |
| ***Cd9*** | **1.0235** |  |  |
| ***Tnfaip8l2*** | **1.0051** |  |  |

adjusted p-value <0.05

**Additional File1: Table S5. Genes that are upregulated in Venn diagrams.**

**Unique genes (199) in CD4+EGFP+ vs. CD4+EGFP- cells**

| Abcg1 | Gid8 | Ms4a4c | Rnaset2 | Zbtb9 |
| --- | --- | --- | --- | --- |
| Abtb2 | Gimap1 | Ms4a8 | Rnf216 | Zc3hav1 |
| Acot2 | Glcci1 | Msl1 | S1pr1 | Zfp281 |
| Agpat5 | Glg1 | Mtdh | Sbk1 | Zfp36l1 |
| Aig1 | Gpcpd1 | Mtmr3 | Scml4 | Zfp830 |
| Aldh2 | Gpr15 | Myh14 | Sell | Znrf2 |
| Ap1s3 | Gpr171 | Mylip | Sesn2 | Zswim4 |
| Arid5b | Gpr174 | Mynn | Sh2d3c |  |
| As3mt | Gsn | Myo10 | Sh3tc1 |  |
| Bex4 | Hebp2 | Neil1 | Siah1 |  |
| Btrc | Herpud2 | Nfatc1 | Sipa1 |  |
| Castor2 | Hexim1 | Nsd3 | Sipa1l1 |  |
| Cblb | Hspb11 | Nupr2 | Slamf7 |  |
| Cbx6 | Iah1 | Ocln | Slc23a3 |  |
| Ccn3 | Id3 | Oga | Slc43a2 |  |
| Ccr5 | Ifnar1 | Pacc1 | Slc5a3 |  |
| Cd27 | Ift80 | Parp12 | Smpd1 |  |
| Cd38 | Igf1r | Pbx4 | Snn |  |
| Cd47 | Ikzf2 | Peli1 | Son |  |
| Cd80 | Il16 | Pgm2l1 | Specc1l |  |
| Cd96 | Il18 | Phf20 | Stap2 |  |
| Cd99l2 | Il1rl1 | Phf8 | Stbd1 |  |
| Cerk | Il2rb | Phyh | Taf7 |  |
| Chn1 | Ing1 | Pias1 | Taok2 |  |
| Clta | Itgb8 | Pias2 | Tbcc |  |
| Cnn3 | Itm2a | Pja2 | Tbl1xr1 |  |
| Cradd | Jak1 | Plac8 | Tent5c |  |
| Crebrf | Kcnk6 | Plagl2 | Tgif1 |  |
| Cyfip1 | Kdsr | Plpp2 | Tigd2 |  |
| Dag1 | Klf7 | Prf1 | Tmem130 |  |
| Daglb | Krt18 | Prkcq | Tmem71 |  |
| Decr2 | Lamp1 | Prkcz | Tnfrsf13b |  |
| Dnmt3a | Ldlrad4 | Psen2 | Tnfrsf1b |  |
| Dph2 | Limd1 | Ptbp3 | Tnip1 |  |
| Dtnb | LOC302022 | Ptpn7 | Tnnt2 |  |
| Dtnbp1 | Lrp11 | Qprt | Tut4 |  |
| Dusp10 | Lsr | Rab11fip1 | Ube2r2 |  |
| Eif2ak1 | Ly6al | Rab19 | Ube3a |  |
| Eif4e3 | Lynx1 | Rab20 | Upb1 |  |
| Epsti1 | Map3k1 | Rab5b | Usp18 |  |
| Eva1b | Mbd2 | Rabgap1l | Usp3 |  |
| Fas | Mbnl2 | Rap1gds1 | Vamp5 |  |
| Fbxo46 | Mbtps1 | Rapgef6 | Vnn1 |  |
| Foxn3 | Mcart1 | Rbl2 | Was |  |
| Foxp1 | Mfap3 | Rdh16 | Wipf1 |  |
| Frmd6 | Mfge8 | RGD1309104 | Zbtb2 |  |
| Gadd45g | Mmrn2 | RGD1563888 | Zbtb4 |  |
| Gbp4 | Mns1 | Rin3 | Zbtb7a |  |

**Unique genes (306) in CD8+EGFP+ vs. CD8+EGFP- Treg**

| Acbd3 | Chd6 | Gadd45b | LOC100911177 | Ppp1r12b | Snrnp48 | Vav3 |
| --- | --- | --- | --- | --- | --- | --- |
| Acin1 | Chkb | Gapt | LOC100912312 | Ppp2r3b | Socs1 | Vcam1 |
| Acrbp | Clasrp | Gdi1 | LOC361985 | Prickle3 | Speg | Vipr2 |
| Adamts10 | Clcf1 | Gfra2 | LOC682870 | Prkd2 | Spic | Vom2r34 |
| Adck5 | Clk1 | Gna15 | Lpin2 | Prr22 | Spindoc | Vps13d |
| Ahsa2 | Col17a1 | Gorasp1 | Lrrc23 | Prrc2a | Spns1 | Vps37c |
| Akap8l | Cpd | Gpat2 | Lrrc71 | Prrt1 | Spry1 | Vps54 |
| Anks1a | Cry2 | Gpat4 | Lrtm2 | Psap | Srrm2 | Vwa7 |
| Anxa3 | Csnk1g2 | Gsdma | Luc7l3 | Ptprv | Ssbp4 | Vwf |
| Apoe | Dap3 | Gtpbp6 | Ly49s7 | Pwwp3a | Sstr3 | Wwc2 |
| Arhgap21 | Dcaf6 | Hap1 | Lyst | Pxdc1 | St3gal5 | Ypel3 |
| Arhgef40 | Dclre1c | Herc2 | Mab21l3 | Pygo2 | Stk38 | Zbtb17 |
| Armc5 | Ddx17 | Hgs | Macf1 | Rab12 | Stox2 | Zc3h7a |
| Arrdc2 | Ddx39b | Hmox1 | Map2k5 | Rab4b | Stra6 | Zcchc7 |
| Arvcf | Def8 | Hpgds | Map4k4 | Ralgds | Stx1a | Zfp347 |
| Ash1l | Dennd1c | Hps5 | Mier2 | Ralgps2 | Supt5h | Zfp641 |
| Atad3a | Dgka | Hsd11b2 | Mink1 | Rapgef2 | Syne4 | Zfpm1 |
| Atp13a1 | Dgkz | Hspbap1 | Mir155hg | Rapgef5 | Tdp2 | Zswim8 |
| Atpaf1 | Dhx30 | Hus1 | Mnt | Rarg | Tfb2m |  |
| Aurkc | Dhx37 | Ift43 | Mon2 | Rarres1 | Tfrc |  |
| Bcl2l1 | Dkc1 | Ikbke | Mrap2 | Rbfox2 | Timp1 |  |
| Bco2 | Dlx4 | Il2 | Mrc1 | Rbpjl | Tincr |  |
| Begain | Dnase1l3 | Il6r | Mtmr12 | Rere | Tmem156 |  |
| Blnk | Dpp4 | Il6st | Mzb1 | Rexo1 | Tmem161a |  |
| Brd8 | Dse | Il9r | Ncf1 | Rftn1 | Tmem176a |  |
| Brf2 | Dtx2 | Inpp4a | Nemf | Rfx1 | Tmem176b |  |
| C1qa | Dus3l | Inpp5b | Neurl3 | RGD1307100 | Tnfrsf11b |  |
| C1qb | Dusp5 | Insl3 | Nr4a1 | RGD1564149 | Tnfrsf25 |  |
| C1qc | Edc4 | Irf4 | Nsf | Ripor1 | Tnk2 |  |
| Cacnb1 | Edrf1 | Itga6 | Nt5dc2 | Rn45s | Tnks |  |
| Cadm4 | Egln2 | Itgb5 | Olig3 | Rrp9 | Tnnc2 |  |
| Camta2 | Emid1 | Itm2c | Pced1b | Rspo2 | Tnnt3 |  |
| Capn5 | Erc1 | Itpr2 | Pdzd4 | Rsrp1 | Tns3 |  |
| Cbx7 | Ercc2 | Jag1 | Phf1 | Rundc3a | Tpcn2 |  |
| Cc2d1b | Ercc6 | Jak3 | Phyhd1 | Runx1 | Traf3ip2 |  |
| Ccdc134 | Faap20 | Jmjd1c | Pik3c2a | Sat1 | Trappc10 |  |
| Ccl1 | Fam117a | Kbtbd11 | Pim3 | Sbf1 | Trim34 |  |
| Ccnd1 | Fam193b | Kcnn4 | Pip4p1 | Sbsn | Trip6 |  |
| Ccnk | Fam214a | Kdm4a | Pitpnm2 | Sdc3 | Trir |  |
| Cd163 | Fam43a | Kdr | Pkib | Sdc4 | Trpm2 |  |
| Cd5l | Fam98c | Kiaa0408L | Pkn1 | Setd4 | Ttc22 |  |
| Cd83 | Fastkd1 | Kif13a | Pla2g2d | Sf3a2 | Ube2h |  |
| Cdh1 | Fcho1 | Krit1 | Plcd1 | Slc17a9 | Ubtf |  |
| Cdk16 | Fcna | Laptm4b | Plod1 | Slc35e4 | Ubxn6 |  |
| Celf5 | Foxi1 | Lats1 | Plpp3 | Slc40a1 | Ulk1 |  |
| Cep95 | Fyttd1 | Ldb1 | Pms2 | Slc4a11 | Usf2 |  |
| Cfd | Fzd8 | Leng8 | Pnn | Smpd2 | Usp30 |  |
| Cfp | Gabbr1 | Lingo4 | Pou2f2 | Smurf2 | Utrn |  |

**Common genes (49) between CD4+EGFP+ vs. CD4+EGFP- cells and CD8+EGFP+**

**vs. CD8+EGFP- cells**

| Abcc5 | Wdr25 |
| --- | --- |
| Abhd17c | Zc3h12d |
| Adora2a |  |
| Aff3 |  |
| Arl5c |  |
| Art2b |  |
| Atp6v0a1 |  |
| Bcl3 |  |
| Bcl9l |  |
| Bex1 |  |
| C1qtnf6 |  |
| Cipc |  |
| Cyp2s1 |  |
| Dst |  |
| Eno2 |  |
| Foxp3 |  |
| Il21r |  |
| Il4r |  |
| Inpp5a |  |
| Irak3 |  |
| Ly6e |  |
| Manba |  |
| Nfkbia |  |
| Nsg1 |  |
| Paqr5 |  |
| Patj |  |
| Pcbp3 |  |
| Pde2a |  |
| Phlda1 |  |
| Pik3ip1 |  |
| Ppfibp1 |  |
| RGD1565410 |  |
| Rhoh |  |
| Rilpl2 |  |
| Scarb2 |  |
| Serpini1 |  |
| Sesn1 |  |
| Sez6 |  |
| Slc12a7 |  |
| Slc41a3 |  |
| Snx20 |  |
| Thrb |  |
| Tiam1 |  |
| Tnfrsf26 |  |
| Tox |  |
| Ttyh3 |  |
| Vipr1 |  |

**Unique genes (256) between CD4+EGFP+ vs. CD8+EGFP+ Treg**

| Abca7 | Cers2 | Gpx4 | Ndufa9 | Rnf125 | Trappc3 |
| --- | --- | --- | --- | --- | --- |
| Abhd4 | Chid1 | Gstm1 | Ndufb11 | Rpain | Trappc4 |
| Acadvl | Chmp3 | Gtf2a2 | Ndufb4 | Rpe | Trat1 |
| Acp1 | Cklf | Hdhd2 | Nfyb | Rps19l2 | Trmt112 |
| Actl6a | Cldnd1 | Hist1h2bq | Nmi | Rsu1 | Tsnax |
| Adi1 | Clec4a3 | Hmgn3 | Nucb1 | RT1-Da | Tspo |
| Adprm | Clic1 | Hsd17b11 | Oas1i | Rtp4 | Txndc17 |
| Aga | Clk4 | Hspa13 | Ormdl2 | Sar1b | Ubb |
| Agk | Cnih4 | Ifi47 | Ost4 | Scp2 | Ubl4a |
| Agtrap | Coa3 | Ifitm2 | Papss1 | Scpep1 | Ubxn8 |
| Alkbh6 | Coa4 | Ift172 | Pcnp | Selenok | Utp11 |
| Alox5ap | Cops2 | Igtp | Pdcd10 | Selplg | Vamp8 |
| Amelx | Cr1l | Il2rg | Pde4b | Sfr1 | Vdac3 |
| Anapc13 | Crcp | Irf7 | Pex16 | Skap2 | Vps29 |
| Anp32a | Cript | Isca2 | Pex19 | Smco4 | Ythdf2 |
| Anxa1 | Cryl1 | Isg15 | Pkm | Smyd3 | Zfp566 |
| Anxa4 | Cst3 | Jpt1 | Pnkd | Snx3 |  |
| Anxa5 | Cstb | Krtcap2 | Pnrc2 | Spcs1 |  |
| Ap2m1 | Ctsb | Leng1 | Ppdpf | Spib |  |
| Aprt | Ctss | LOC100125368 | Ppil3 | Spsb2 |  |
| Arf4 | Cyba | LOC100302372 | Ppp1r11 | Ssr3 |  |
| Arhgap15 | Daam1 | LOC100910973 | Ppp2r5c | Ssr4 |  |
| Arhgdib | Dctn6 | LOC688090 | Prdx2 | Stom |  |
| Arpp19 | Ddost | Lrpap1 | Prdx4 | Strada |  |
| Atp5f1c | Dguok | Lsm10 | Prr13 | Stt3a |  |
| Atp5if1 | Dnajc19 | Ly86 | Prrt3 | Stub1 |  |
| Atp5pb | Dusp6 | Lyc2 | Psenen | Stx18 |  |
| Atp5pf | Ech1 | Mal | Psmb7 | Sugt1 |  |
| Atp6v0e1 | Eef1akmt1 | Mapre1 | Psmd1 | Sumo1 |  |
| Avpi1 | Eef1e1 | Med10 | Psmd10 | Sv2a |  |
| Bcap31 | Emd | Med19 | Psmd11 | Szrd1 |  |
| Bccip | Erg28 | Mien1 | Psmd12 | Taf9b |  |
| Bpgm | Erp44 | Mpc1 | Psmd14 | Tapbp |  |
| Bud31 | Evi2a | Mpc2 | Rab11b | Tcea1 |  |
| Calm1 | Exoc6 | Mrpl18 | Rab2a | Tceal8 |  |
| Calm2 | Fcnb | mrpl24 | Rab8b | Tcf4 |  |
| Camk4 | Fermt3 | Mrpl34 | Rab9a | Tgoln2 |  |
| Capg | Fkbp3 | Mrpl50 | Rabac1 | Tmbim4 |  |
| Ccl6 | Frg1 | Mrps12 | Rbks | Tmbim6 |  |
| Ccr6 | Fubp1 | Mrps16 | Rdh8 | Tmco1 |  |
| Cd19 | Gabarap | Ms4a1 | RGD1306063 | Tmed10 |  |
| Cd2 | Galm | Mthfs | RGD1359127 | Tmem128 |  |
| Cd300c2 | Gda | Naa38 | RGD1359508 | Tmem179b |  |
| Cd4 | Gemin7 | Naa60 | RGD1559482 | Tmem205 |  |
| Cd48 | Glmp | Nagk | RGD1563941 | Tmem50a |  |
| Cd53 | Gm2a | Napsa | Rhno1 | Tmem59 |  |
| Cd74 | Gmfg | Ncoa4 | Rida | Tnfaip8l2 |  |
| Cdc123 | Gpx1 | Ndufa13 | Rnasek | Trappc1 |  |

**Common genes (48) between CD4+EGFP+ vs. CD8+EGFP+ cells and CD4+EGFP+**

**vs. CD4+EGFP- cells**

| Acp5 |
| --- |
| Ak3 |
| Apobec2 |
| Bnip3l |
| Cd9 |
| Cdkn1b |
| Cdkn2c |
| Ctsc |
| Ctse |
| Cyb5r3 |
| Ethe1 |
| Gata3 |
| Gimap7 |
| Glrx |
| Grb14 |
| Hps6 |
| Icos |
| Ifngr1 |
| Ipcef1 |
| Lbh |
| Lrrc8c |
| MGC105567 |
| Mgst1 |
| Ms4a6bl |
| Mxd4 |
| Ncr3 |
| Nmnat1 |
| Nmur1 |
| Nrp1 |
| Phlda2 |
| Pja1 |
| Prxl2b |
| Ptafr |
| Ptp4a2 |
| Pts |
| Pvr |
| Rab25 |
| Sec11c |
| Serpinb6a |
| Shisa5 |
| Smagp |
| Snx2 |
| Sri |
| Tank |
| Tmem140 |
| Traf3ip3 |
| Wls |
| Zfp148 |

**Common genes (5) between CD4+EGFP+ vs. CD8+EGFP+ and CD8+EGFP+ vs. CD8+EGFP- cells**

| Cd79b |
| --- |
| Cyb561a3 |
| Lgmn |
| Lta |
| Ramp3 |

**Common genes (13) between CD4+EGFP+ vs. CD8+EGFP+ and CD8+EGFP+ vs. CD8+EGFP-**

**and CD4+EGFP+ vs. CD4+EGFP- cells**

| Apbb1 |
| --- |
| Ctsl |
| Fcmr |
| Il2ra |
| Lrrc32 |
| Maf |
| Prtfdc1 |
| Samhd1 |
| Selenop |
| Smpdl3a |
| Tnfrsf18 |
| Tnfrsf4 |
| Tnfrsf9 |

**Additional File1: Table S6. Genes that are downregulated in Venn diagrams.**

**Unique genes (239) in CD4+EGFP+ vs. CD4+EGFP- cells**

| Adh6 | Espn | Lingo4 | Pgam1 | Shisa8 |
| --- | --- | --- | --- | --- |
| Akr1e2 | Etv6 | Lmf2 | Pip5k1b | Slc11a1 |
| Ankrd13d | Exog | Lmna | Plaur | Slc22a4 |
| Ankrd39 | Exosc2 | LOC100910945 | Plk3 | Slc37a3 |
| Apoo | F8 | LOC286960 | Poc1a | Smc5 |
| Arntl | Fabp5 | LOC312273 | Pola2 | Smim3 |
| Atad2 | Fam111a | LOC361914 | Pomt1 | Snrpa |
| Atf6 | Fam136a | LOC365238 | Ppa1 | Sorcs3 |
| Atp10a | Fbxo41 | LOC498265 | Ppp1r14b | Sox17 |
| Atp2b4 | Fhad1 | Lsm2 | Ppp1r9b | Specc1 |
| Baiap2l1 | Fkbp11 | Map3k6 | Pprc1 | Sphk1 |
| Bik | Foxm1 | Mboat1 | Prdm8 | St3gal5 |
| Bpifb4 | Fut7 | Mgat4a | Prmt5 | ST7 |
| Capn10 | Fyco1 | Mir155hg | Prpf19 | Sv2b |
| Ccl3 | Gar1 | Mpp4 | Prr5l | Sytl1 |
| Ccr6 | Gch1 | Mrpl12 | Psat1 | Tbl3 |
| Cd226 | Gcsh | Mrpl38 | Ptn | Tfb1m |
| Cd40lg | Gem | Mrto4 | Pus7 | Tfdp1 |
| Cd99 | Ggt1 | Mtfr2 | Pxdc1 | Tgm3 |
| Cdca7 | Glod5 | Mvd | Qtrt1 | Thg1l |
| Cenpn | Gna14 | Myadml2 | Rab38 | Thop1 |
| Cfap45 | Gpatch4 | Myom3 | Rad23a | Tigar |
| Chd7 | Gpr146 | N5 | Rad51 | Timd2 |
| Ciapin1 | Gsdma | Naglt1 | Rap1gap2 | Timm9 |
| Ckb | Herpud1 | Nat10 | Rapgef2 | Tma16 |
| Clcn4 | Hspd1 | Nck2 | Rbm28 | Tmem97 |
| Cldn10 | Hyou1 | Ncl | Rbpj | Tnf |
| Clspn | Ier3 | Ncln | Rcc1l | Tnfrsf21 |
| Cluh | Ifitm1 | Nfasc | Resp18 | Tnfrsf25 |
| Cnr2 | Ifrd2 | Nkx6-2 | Rfc3 | Tpi1 |
| Cntd1 | Igflr1 | Nme2 | Rflna | Trim2 |
| Col14a1 | Igsf9 | Nmral1 | Rflnb | Tsga10ip |
| Col4a1 | Il17f | Noc4l | RGD1308065 | Tufm |
| Cpm | Il17rc | Nolc1 | RGD1309350 | Txlng |
| Ctps1 | Il17re | Nop9 | RGD1310852 | Ube2s |
| Cyp4f17 | Il3ra | Nr1d1 | RGD1560289 | Uck2 |
| Dclre1b | Kcng4 | Nthl1 | RGD1564149 | Usp36 |
| Dctpp1 | Kcnk1 | Nup93 | RGD1564664 | Usp44 |
| Dear | Kif5c | Olah | Rhbdd3 | Vars1 |
| Depdc1 | Klhl8 | Olfm1 | Rhbdf2 | Vegfb |
| Dnajb7 | Klrc1 | Pa2g4 | Rpf2 | Vwa7 |
| Dnajc11 | Knop1 | Pak3 | Rps6ka2 | Xcl1 |
| Dync2i2 | Kntc1 | Pcdhb2 | S100a4 | Ybey |
| Ecm1 | Lama3 | Pde11a | S100a5 | Ydjc |
| Ehhadh | Lap3 | Pde7a | Scg5 | Zbtb8a |
| Elmod2 | Large1 | Pelp1 | Sdf2l1 | Zfp707l1 |
| Emb | Lat | Pex1 | Sh3bp5 | Zfp9 |
| Enpp3 | Lgals3 | Pex11b | Shisa3 |  |

**Additional File1: Table S6. Unique genes (333) in CD8+EGFP+ vs. CD8+EGFP- Treg**

| Abracl | Cd81 | Fcnb | Larp7 | Nudt1 | Rdh8 | Stt3a |
| --- | --- | --- | --- | --- | --- | --- |
| Acat1 | Cdc123 | Fen1 | Lck | Nudt21 | Rfc4 | Stx11 |
| Acp1 | Cdc20 | Fxyd5 | Ldhb | Nusap1 | RGD1359127 | Sub1 |
| Actg1 | Cdc25b | Fyn | LOC102554317 | Nxt2 | RGD1359508 | Suclg1 |
| Actr3 | Cdca8 | Gale | LOC688090 | Oprd1 | Rpa3 | Susd2 |
| Agk | Cdhr1 | Galm | Lsm10 | Ost4 | Rpain | Svbp |
| Agpat4 | Cdk4 | Gas7 | Lsm12 | Ostf1 | Rpe | Szrd1 |
| Aifm1 | Cdkn3 | Gbp2 | Ly49s3 | Ostm1 | Rps19l2 | Tagln2 |
| Ak2 | Cenpm | Gemin7 | Ly49s6 | Oxr1 | Rps27l | Tapbp |
| Ak8 | Cers2 | Gimap4 | Lyc2 | Pabpc1 | Rrm1 | Tcea1 |
| Anapc13 | Cfl1 | Gins1 | Mad2l1 | Papss1 | Rrm2 | Tcf19 |
| Anxa2 | Chchd5 | Glrx2 | Magohb | Pcnp | Rsu1 | Tent5a |
| Anxa6 | Cisd3 | Gmfg | Mapre1 | Pdcd1 | RT1-Da | Tes |
| Arpc1b | Clic1 | Gnai3 | Mapre2 | Pde4b | RT1-Db1 | Tgoln2 |
| Arpc5 | Cnrip1 | Gpr68 | Mbd2 | Pgk1 | RT1-DMa | Timm17b |
| Asf1b | Cops2 | Gyg1 | Mcm4 | Phf11 | RT1-DMb | Timm23 |
| Atp5f1a | Cox5b | Gzma | Mdh1 | Phf11b | Rtp4 | Tk1 |
| Atp5f1b | Cox6b1 | Gzmk | Med19 | Pik3cg | S100a10 | Tmbim6 |
| Atp5f1c | Crcp | H2ax | MGC105649 | Pkm | S100a9 | Tmed2 |
| Atp5pb | Cst7 | H2az1 | Mkrn1 | Plaat3 | S1pr5 | Tmem126a |
| Atp5pf | Cuedc2 | H2bc12 | Mpc1 | Plek | Samd3 | Tmsb15b2 |
| Aurka | Cx3cr1 | Haus4 | Mpst | Plk4 | Sar1b | Tnfsf9 |
| Aurkb | Cxcr3 | Hcst | Mrnip | Pnrc2 | Sema4a | Tpgs2 |
| B4galt5 | Cxcr6 | Hdgf | Mrpl18 | Pole | Serbp1 | Tprg1l |
| Banf1 | Cyria | Hikeshi | Mrpl34 | Pop4 | Serpinb9 | Tpst2 |
| Bcap29 | Dapk2 | Hltf | Mthfd1 | Ppib | Sh2d1a | Traf1 |
| Becn1 | Dars2 | Hmgb1 | Myl12a | Ppil3 | Sh2d2a | Trappc1 |
| Birc5 | Dbi | Hmgb3 | Mylpf | Ppp2r5c | Sh3bgr | Tubb4b |
| Borcs8 | Def6 | Hmgcs1 | Naa38 | Prdx4 | Sh3bgrl3 | Txn1 |
| Calm1 | Dhcr7 | Hopx | Naa60 | Prim2 | Siva1 | Ube2a |
| Calm2 | Dhfr | Hsd11b1 | Nans | Prkcb | Skap1 | Ubl4a |
| Cap1 | Dmrtc2 | Hsd17b11 | Ncald | Prmt2 | Slamf7 | Uchl3 |
| Casp3 | Dnajc19 | Hspa13 | Ncr1 | Prrt3 | Slbp | Uchl5 |
| Ccl5 | Dstn | Idh2 | Ndufa10 | Psma5 | Slc25a5 | Uqcrfs1 |
| Ccl6 | Dynlt1 | Idh3a | Ndufa2 | Psmb8 | Slc39a8 | Uxt |
| Ccnb1 | E2f1 | Isca2 | Ndufa9 | Psmd1 | Slc9a3r1 | Vdac3 |
| Ccnb2 | Eef1akmt1 | Isg15 | Ndufb11 | Psmd11 | Slirp | Vim |
| Ccnd3 | Eef2 | Itgb2 | Ndufb4 | Psmd12 | Spcs2 | Xpo1 |
| Ccng1 | Eif4e2 | Itm2b | Ndufb6 | Psmd14 | Spink2 | Xrcc5 |
| Ccr5 | Eipr1 | Jpt1 | Ndufb9 | Psmf1 | Spint1 | Ybx3 |
| Cd164 | Emd | Kif20a | Ndufs1 | Pxn | Spn | Yif1b |
| Cd2 | Emp1 | Kif22 | Ndufs3 | Pycard | Spr | Ythdf2 |
| Cd38 | Emp3 | Klra22 | Ngp | Rab11a | Sptssa | Ywhae |
| Cd3e | Erp44 | Klrb1a | Nicn1 | Rab27a | Ssr2 | Ywhaq |
| Cd3g | Esd | Klre1 | Nmi | Rab2a | Ssr3 | Zbtb32 |
| Cd53 | Etfb | Knstrn | Nptn | Rab3d | St3gal6 |  |
| Cd59 | Evi2b | Lage3 | Nt5c | Rab9a | Stmn1 |  |
| Cd74 | Fam89b | Lamp2 | Nucb1 | Rbms1 | Strada |  |

**Additional File1: Table S6. Common genes (24) between CD4+EGFP+ vs. CD4+EGFP- cells and CD8+EGFP+ vs. CD8+EGFP- cells**

| Ahnak |
| --- |
| Amt |
| Cdc42ep3 |
| Chchd6 |
| Chek1 |
| Ctla2a |
| Faslg |
| Gapdh |
| Gzmb |
| Gzmc |
| Id2 |
| Klrk1 |
| Lgals1 |
| Lgals3bp |
| Mcm2 |
| Myadm |
| Myo1f |
| Nkg7 |
| Pcp4 |
| Pwp1 |
| Ran |
| S100a6 |
| Tmem107 |
| Tmem204 |

**Additional File1: Table S6. Unique genes (212) between CD4+EGFP+ vs. CD8+EGFP+ Treg**

| Acap1 | Cstf3 | Leng8 | Rbm25 | Ttc3 |
| --- | --- | --- | --- | --- |
| Acap3 | Cxcl17 | LOC100911177 | Rbm34 | Uap1l1 |
| Acbd3 | Cxxc5 | LOC100912312 | Rcn1 | Ubtf |
| Acin1 | Cyth4 | LOC682870 | Recql5 | Ubxn6 |
| Acrbp | Dcaf6 | Lrrc42 | Rev1 | Uchl1 |
| Adamts10 | Defa5 | Luc7l2 | Rev3l | Unc119b |
| Adcy6 | Dhx30 | Luc7l3 | RGD1307100 | Usp11 |
| Adgrg5 | Dhx37 | Ly49i4 | RGD1560108 | Usp38 |
| Agpat3 | Dhx9 | Ly49i5 | Rnf130 | Usp40 |
| Akap2 | Dis3l2 | Ly49s7 | Rnf187 | Vav3 |
| Akap8 | Dnase1l3 | Map2k5 | Rpsa | Vom2r34 |
| Alms1 | Doc2g | Map3k11 | Rsrp1 | Vps13a |
| Ankrd44 | Dock5 | Mast3 | Rundc3a | Vps37c |
| Ap5z1 | Dtx2 | Med15 | Safb2 | Wdr3 |
| Apoe | Dusp2 | Mettl3 | Senp3 | Wdr5 |
| Arf5 | Ehbp1l1 | Mex3b | Sh2b1 | Zbtb17 |
| Arglu1 | Elac2 | Mink1 | Sh2d4a | Zc3h7a |
| Arhgef2 | Emid1 | Mms19 | Shkbp1 | Zfp39 |
| Arhgef7 | Eri1 | Mnt | Slc16a6 | Zfp445 |
| Armc5 | Esrra | Mon2 | Slc25a38 | Zpbp2 |
| Ascc3 | Fam13b | Myef2 | Slc30a5 |  |
| Atf6b | Fam193b | Nat8b | Slc43a1 |  |
| Atp13a1 | Fam98c | Nemf | Slc4a11 |  |
| Bag1 | Fastkd1 | Np4 | Slc7a6 |  |
| Bak1 | Fcgbp | Nrros | Slc9a1 |  |
| Baz1b | Fcna | Nsun2 | Slfn13 |  |
| Bin2 | Gabpb1 | Nt5dc2 | Snorc |  |
| Brf1 | Glrx5 | Nufip1 | Snrnp48 |  |
| C1qa | Gnptg | Pabpc2 | Sorl1 |  |
| C1qb | Gpsm1 | Pabpn1 | Sort1 |  |
| C1qc | Grk6 | Pced1a | Spic |  |
| C2cd2l | Gtpbp10 | Pcm1 | Spindoc |  |
| Cacnb1 | Gtpbp2 | Pik3cd | Sppl2b |  |
| Camta2 | Hip1r | Plekhg5 | Srsf1 |  |
| Cc2d1b | Hmox1 | Pnn | Srsf2 |  |
| Ccdc102a | Hnrnph1 | Pnpla7 | Ssbp4 |  |
| Ccnk | Hps5 | Polr1d | Stk38 |  |
| Cd8a | Hsd11b2 | Ppfia4 | Supt5h |  |
| Cdh1 | Hsd17b8 | Ppp6r1 | Sytl3 |  |
| Cep85 | Insl3 | Prcc | Tap1 |  |
| Cep95 | Itpr3 | Prr7 | Tbcd |  |
| Cfd | Jak3 | Ptms | Tfb2m |  |
| Clasrp | Kdm2a | Pwwp3a | Tmem160 |  |
| Clcf1 | Kdm4a | Pygo2 | Tnk2 |  |
| Clstn1 | Klhdc3 | Rab4b | Trafd1 |  |
| Cnot1 | Klri1 | Rabggtb | Trim39 |  |
| Cnot10 | Lag3 | RatNP-3b | Trim41 |  |
| Cpsf4 | Ldb1 | Rbm17 | Trir |  |

**Additional File1: Table S6. Common genes (24) between CD4+EGFP+ vs. CD8+EGFP+ cells**

**and CD4+EGFP+ vs. CD4+EGFP- cells**

| Abcg3l2 |
| --- |
| Agap2 |
| Arrb2 |
| Ccl1 |
| Cd7 |
| Csnk1g2 |
| Dapk3 |
| Dkc1 |
| Fus |
| Gsto1 |
| Il17ra |
| Il2 |
| Lmbrd2 |
| Mcoln2 |
| Mical1 |
| Ncbp1 |
| Pcyox1l |
| Pstpip1 |
| Rangap1 |
| Rrp9 |
| Stau2 |
| Tbx21 |
| Tmem273 |
| Usp46 |

**Additional File1: Table S6. Common genes (3) between CD4+EGFP+ vs. CD8+EGFP+**

**and CD8+EGFP+ vs. CD8+EGFP- cells**

| Cd8b |
| --- |
| Clic5 |
| Gzmm |

**Additional File1: Table S6. Common genes (3) between CD4+EGFP+ vs. CD8+EGFP+**

**and CD8+EGFP+ vs. CD8+EGFP- and CD4+EGFP+ vs. CD4+EGFP- cells.**

| Ctsw |
| --- |
| Klrd1 |
| Tacc2 |

**Additional File1: Table S7. Increase in different EGFP+ and EGFP- cell subsets in blood following IL-2 treatment.**

|  |  |  | **CD3+ CD4+ EGFP+** | **CD3+CD4+ EGFP-** | **CD3+CD8+ EGFP+** | **CD3+CD8+ EGFP-** | **CD161+EGFP+** | **CD161+EGFP-** |
| --- | --- | --- | --- | --- | --- | --- | --- | --- |
| **Blood** | **Untreated n=4** | **% of total cells** | 5.6 ± 1.4 | 94.9 ± 0.3 | 2.2 ± 0.4 | 96 ± 0.3 | 0 | 2 ±1.4 |
|  |  | **N°cellsx10^5/^ml** | 0.08 ±0.06 | 1.7 ± 0.6 | 0.016 ±  0.005 | 0.7 ± 0.3 | 0 | 0.06 ± 0.04 |
|  | **hIL2 treated n=3** | **% of total cells** | 17.1 ± 0.6 | 82.6 ± 0.4 | 9.8 ± 0.9 | 89.7 ± 0.9 | 0 | 2,7 ± 1.1 |
|  |  | **N°cellsx10^5^ /ml** | 1.5 ± 0.5 | 7.5 ± 2 | 0.5 ± 0.1 | 4.4 ± 1.5 | 0 | 0.6 ± 0.2 |
|  |  | **Fold induction*** | 19 | 4.4 | 31 | 6.3 | 0 | 10 |

* Fold induction of absolute numbers of cells in the IL-2-treated vs. untreated animals

**Additional File1: Table S8. Antibodies used in the study.**

| **Marker** | **Clone** | **Provider** |
| --- | --- | --- |
| Viability Dye 506 |  | Invitrogen eBiosciences |
| Biotin-conjugated mouse anti-rat CD3 | G4.18 | BD Biosciences |
| BV421-conjugated mouse anti-rat CD3 | 1F4 | BD Biosciences |
| PercP-conjugated mouse anti-rat TCRαβ | R7.3 | BD Biosciences |
| PECy7-conjugated mouse anti-rat CD4 | OX35 | BD Biosciences |
| BV421-conjugated mouse anti-rat CD25 | OX39 | BD Biosciences |
| APC-conjugated mouse anti-rat CD161 | 3.2.3 | Produced in house |
| APC-conjugated mouse anti-rat CD8a | OX8 | Produced in house |
| PE-conjugated mouse anti rat CD45RC | OX22 | BD Biosciences |
| PE-conjugated anti-Foxp3 | FJK-16s | Invitrogen eBiosciences |
| PE-conjugated mouse anti-Stat5 | pY694 | BD Biosciences |
| PE-conjugated mouse anti-rat RT1B | OX6 | BD Biosciences |
| PE-conjugated mouse anti-rat CD28 | CD28 | BD Pharmingen |
| PE-conjugated hamster anti-mouse CD27 | LG.3A10 | BD Biosciences |
| BV711-conjugated mouse anti-rat CD44 | OX49 | BD Biosciences |
| BV421-conjugated hamster anti-rat CD62L | HRL1 | BD Biosciences |
| Purified mouse anti-rat CD71 | OX26 | Produced in house |
| Biotin-conjugated mouse anti-rat CD5 | OX19 | Produced in house |
| Purified mouse anti-rat CD26 | OX61 | Produced in house |
| Purified rabbit anti- rat CX3CR1 | TP501 | Produced in house |
| PE-conjugated mouse anti-rat CD106 | MR106 | BD Biosciences |
| PE-conjugated mouse anti-rat CD80 | 3H5 | BD Biosciences |
| Purified mouse anti-rat CD38 | 14.27 | Biolegend |
| PE-conjugated or BV421-conjugated mouse anti-human CD137 | 4BB1 | BD Biosciences |
| Streptavidin APC-Cy™7 |  | BD Biosciences |
| AF568 goat anti-mouse IgG2a |  | Thermofisher |
| R-Phycoerythrin AffiniPure F(ab')₂ Fragment Donkey Anti-Rabbit IgG (H+L) |  | Jackson Immunoresearch |
